# Supplementary material for: Total added sugar consumption is not significantly associated with risk for prediabetes among U.S. adults: National Health and Nutrition Examination Survey, 2013–2018
Source: PLoS One. 2023 Jun 20;18(6):e0286759. doi: 10.1371/journal.pone.0286759 (PMC10281581; doi:10.1371/journal.pone.0286759)
Supplement: S1 File — (DOCX) [file pone.0286759.s001.docx]

**S1 Appendix**

**Material and methods**

**Prediabetes assessment**

The outcome variable for this study was prediabetes which has been previously defined and was based on the American Diabetes Association “Standards of Medical Care in Diabetes - 2021” classification [1]. As part of the NHANES data collection process, physical measurements and laboratory tests are collected during the mobile examination center visit from participants ages ≥12 years [2]. Data files of whole blood specimens of glycohemoglobin (i.e., HbA1c) and FPG were included for analysis in this study to define prediabetes [3, 4]. NHANES collects samples at the examination center using a Tosoh G8 Automated Glycohemoglobin (HbA1c) and Cobas c311 Analyzer (FPG). Detailed NHANES laboratory procedures are reported elsewhere [3, 4].

**Covariates**

Regression models included the following covariate categories: age, gender, race/ethnicity, BMI (kg/m^2^), usual intake for total calories (kcals), physical activity, smoking status, educational attainment, and income.

Demographic variables collected during the NHANES in-home interview included age, gender, and race/ethnicity which were self-reported [5, 6]. Age was reported in years and calculated using participant’s dates of birth. Participants ≥80 years were coded as ‘80’ to prevent risk of participant disclosure. Gender was classified as either “male” or “female” [5]. Race and Hispanic origin was categorized into non-Hispanic White, non-Hispanic Black, Hispanic (including Mexican Americans and Latinos), Asian Americans, or Other Race (including persons not identifying with the previously reported categories) [6, 7]. BMI was categorized using the following CDC classifications for adults: underweight (18.5 kg/m^2^), healthy weight (18.5-24.9 kg/m2), overweight (25-29.9 kg/m^2^), or obese (≥ 30 kg/m^2^) [8]. Health behaviors and sociodemographic factors (physical activity, smoking status, education, and ratio of family income to poverty) were based on self-reported questionnaire data. Physical activity was classified using the NHANES physical activity questionnaire (PAQ650 and PAQ665) and was defined as (yes/no) engagement in ≥10 minutes of moderate and/or vigorous recreational activity during a typical week [9]. Smoking status was defined as either current smoker (tobacco use within the last 5 days) or non-smoker (no reported use within last 5 days) [10]. Education level was defined as having either less than a high school degree, having a high school degree or GED, or having more than a high school degree [6]. Ratio of family income to poverty was taken from the NHANES demographic questionnaire and was categorized using the family monthly poverty level index categories calculated by NHANES (≤1.30, >1.30 to 1.85, and >1.85) which represents common poverty guideline percentages [11].

An additional adjusted model was included to assess for any potential confounding effects of four additional dietary covariates: dietary fiber (gm), total fat (gm), saturated fat (i.e., total saturated fatty acids; gm), and total antioxidant capacity (TAC). All four dietary variables are collected as part of the NHANES dietary assessment which uses the 24-hour dietary recall method [12] and can be found in the NHANES “Dietary Interview- Total Nutrient Intakes” files [DR1TOT, DR2TOT]. The TAC was calculated using the validated equation developed by Floegel [13] and has previously been used to assess TAC in U.S. adults ≥20 years who participated in the NHANES survey [14]. The TAC equation included 8 antioxidative vitamins: vitamin A RAE (mcg), vitamin C (mg), vitamin E as alpha-tocopherol (mg), α-carotene (mcg), β-carotene (mcg), β-cryptoxanthin (mcg), lycopene (mcg) and lutein-zeaxanthin (mcg) which were all available in the NHANES “Dietary Interview- Total Nutrient Intakes” files [DR1TOT, DR2TOT].

Dietary fiber, total fat, saturated fat, and TAC were analyzed using the same methods previously described in the main manuscript. A survey weighted logistic regression model that adjusted for age in years, gender, race/ethnicity, BMI (kg/m^2^), total energy intake (kcal/day), engagement in physical activity, smoking status, education level, and family poverty to income ratio (PIR), dietary fiber, total fat, saturated fat, and TAC was tested to assess whether usual intake of total and percent added sugar intakes were associated with an increased odds of prediabetes relative to normoglycemia.

| **Supplemental Table 1. A**djusted odds of prediabetes for total added sugar (g^a^) in U.S. adults ≥20 years with normoglycemia and prediabetes, the NHANES^b^ 2013-2018 | | | | | | |
| --- | --- | --- | --- | --- | --- | --- |
|  |  | **Estimate**^c^ | **SE**^d^ | ***p* value** | **OR**^e^ | **95% CI**^f^ |
| **Adjusted** (N=4,617)^g,h^ | Intercept | -2.88 | .38 | <.01 | - | - |
|  | Total added sugar (g) | .0001 | .001 | .59 | 1.00 | .99-1.00 |
| Age (years) |  | .04 | .003 | <.01 | 1.04 | 1.04-1.05 |
| Gender | Female (Ref.^i^) | - | - | - | - | - |
|  | Male | .662 | .118 | <.01 | 1.94 | 1.53 -2.46 |
| Race and ethnicity | Non-Hispanic White (Ref.) | - | - | - | - | - |
|  | Non-Hispanic Black | .124 | .111 | .27 | 1.13 | .91-1.42 |
|  | Hispanic (including Mexican American and Latino) | .409 | .123 | <.01 | 1.51 | 1.17-1.93 |
|  | Asian American | .850 | .135 | <.01 | 2.34 | 1.78-3.07 |
|  | Other Race | .754 | .214 | <.01 | 2.13 | 1.38-3.27 |
| BMI^j^ | Underweight ≤ 18.49 kg/m2 (Ref.) | - | - | - | - | - |
|  | Normal 18.5-24.99 kg/m2 | .024 | .210 | .91 | 1.02 | .67-1.56 |
|  | Overweight 25-29.99 kg/m2 | .625 | .252 | .02 | 1.87 | 1.12-3.10 |
|  | Obese ≥30 kg/m2 | 1.13 | .236 | <.01 | 3.10 | 1.93-4.98 |
| Total calorie intake (kcal^k^ /day) |  | .00036 | .0002 | .07 | 1.00 | 0.99-1.00 |
| Moderate and/or vigorous physical activity^l^ | No (Ref.) | - | - | - | - | - |
|  | Yes | -.163 | .10 | .12 | .85 | .69-1.04 |
| Smoking status^m^ | Smoker (Ref.) | - | - | - | - | - |
|  | Non-smoker | -.046 | .140 | .74 | .96 | .72-1.27 |
| Education^n^ | < High school degree (Ref.) | - | - | - | - | - |
|  | High school degree | .259 | .150 | .09 | 1.30 | .96-1.75 |
|  | > High school degree | -.047 | .149 | .73 | .95 | .71-1.29 |
| PIR^o^ | <1.3 (Ref.) | - | - | - | - | - |
|  | 1.3-1.85 | .125 | .126 | .32 | 1.13 | .88-1.46 |
|  | >1.85 | .008 | .136 | .96 | .99 | .75-1.31 |
| Dietary fiber |  | .003 | .014 | .85 | 1.00 | .98-1.03 |
| Total fat |  | .002 | .006 | .73 | 1.02 | .99-1.02 |
| Saturated fat |  | .025 | .014 | .09 | 1.03 | .99-1.06 |
| Vitamin antioxidant capacity |  | -.002 | .016 | .90 | .99 | .97-1.03 |
| ^a^g = grams  ^b^NHANES = National Health and Nutrition Examination Survey  ^c^Estimate (β Coefficient) for usual intake of added sugar (g) represents a change in the odds of having prediabetes for every 1-gram increase in added sugar.  ^d^SE = standard error  ^e^OR = odds ratio  ^f^CI = confidence interval  ^g^Model fit- C statistic: .733. Ns between unadjusted and adjusted models differ due to missing covariates included in the adjusted model.  ^h^Covariates included in the adjusted models are age, gender, race/ethnicity, BMI, total calorie intake (kcal/day), moderate and/or vigorous physical activity, smoking status, education, family income to poverty ratio (PIR).  ^i^Ref = reference category  ^j^BMI = body mass index and was based on standard weight status categories using CDC criteria for underweight, normal weight, overweight, obese.  ^k^kcal = kilocalories NHANES  ^l^Physical activity was based on self-reported data of participant engagement in ≥10 minutes of moderate or vigorous recreational activity during a typical week (yes/no)  ^m^Smoking status was based on self-reported data about the use of tobacco products within the last 5 days (yes/no). Smoker was defined as using products within the last 5 days. Non-smoker was defined as no use of tobacco products within the last 5 days.  ^n^Education was based on self-reported data asking participants their highest grade or level of school completed/received. < High school includes less than high school degree or no high school diploma. High school includes being a graduate or having a GED or equivalent. > High school includes some college/ associate degree or greater.  ^o^PIR = family poverty to income ratio. <1.3 indicates below PIR, 1.3-1.85 indicates at or above PIR, and > 1.85 indicates above PIR. | | | | | | |

| **Supplemental Table 2.** Estimated risk of prediabetes at mean and tertials of total added sugar (g^a^) in U.S. adults ≥20 years, the NHANES^b^ 2013-2018 | | | | | |
| --- | --- | --- | --- | --- | --- |
|  | **Total Added Sugar** | **Estimate**^c^ | | **SE**^d^ | |
| **Adjusted**^e,f^ | Mean (73g) | .613 | .018 | |  |
| Prediabetes (N=2,735) | 1st Q^g^ (43g) | .608 | .020 | |  |
|  | Median Q (64g) | .611 | .018 | |  |
|  | 3rd Q (93g) | .616 | .018 | |  |
| ^a^g = grams  ^b^NHANES = National Health and Nutrition Examination Survey  ^c^Estimates represent the risk probability for prediabetes based on mean and tertial intakes of total added sugar (g/day).  ^d^SE = standard error  ^e^Model fit- C statistic: .733 and p-value = .59.  ^f^Adjusted for age, gender, race/ethnicity, BMI, total calorie intake (kcal/day), moderate and/or vigorous physical activity, smoking status, education, family poverty to income ratio (PIR), dietary fiber, total fat, saturated fat, and vitamin antioxidant capacity.  ^g^Q = quartiles | | | | | |

**Supplementary References**

1. American Diabetes Association. 2. Classification and diagnosis of diabetes: standards of medical care in diabetes—2021. Diabetes Care. 2021;44(Supplement 1):S15. doi: 10.2337/dc21-S002.

2. Zipf G, Chiappa M, Porter KS, Ostchega Y, Lewis BG, Dostal J. National Health and Nutrition Examination Survey plan and operations, 1999-2010. Vital and Health Statistics. 2013;1(56). Epub 1.

3. Centers for Disease Control and Prevention. National Center for Health Statistics. NHANES 2013-2014 Laboratory Methods- Laboratory Procedure Manual: Glycohemoglobin n.d. [cited 2019 March 28]. Available from: <https://wwwn.cdc.gov/nchs/data/nhanes/2013-2014/labmethods/GHB_H_MET_GLYCOHEMOGLOBIN.pdf>.

4. National Health and Nutrition Examination Survey. Plasma Fasting Glucose Laboratory Procedure Manual: Centers for Disease Control and Prevention; 2020 [cited 2022 February 23]. Available from: <https://wwwn.cdc.gov/nchs/data/nhanes/2017-2018/labmethods/GLU-J-MET-508.pdf>.

5. 2013-2014 Demographics Variable List [Internet]. [cited January 5, 2022]. Available from: <https://wwwn.cdc.gov/nchs/nhanes/search/variablelist.aspx?Component=Demographics&Cycle=2013-2014>.

6. 2017-2018 Demographic Variables and Sample Weights (DEMO_J) [Internet]. Centers for Disease Control and Prevention. 2020 [cited January 1, 2021]. Available from: <https://wwwn.cdc.gov/Nchs/Nhanes/2017-2018/DEMO_J.htm>.

7. Centers for Disease Control and Prevention. National Center for Health Statistics. National Health and Nutrition Examination Survey (NHANES): Analytic Guidelines, 2011-2016: Centers for Disease Control and Prevention; 2018 [cited 2019 February 22]. Available from: <https://wwwn.cdc.gov/nchs/nhanes/analyticguidelines.aspx#analytic-guidelines>.

8. Centers for Disease Control and Prevention. About Adult BMI: U.S. Department of Health and Human Services; 2020 [cited 2020 August 27]. Available from: <https://www.cdc.gov/healthyweight/assessing/bmi/adult_bmi/index.html>.

9. 2017-2018 Physical Activity (PAQ_J) [Internet]. Centers for Disease Control and Prevention. [cited January 2021]. Available from: <https://wwwn.cdc.gov/Nchs/Nhanes/2017-2018/PAQ_J.htm#PAQ650>.

10. 2017-2018 Smoking- Cigarette Use (SMQ_J) [Internet]. Centers for Disease Control and Prevention. 2020 [cited January 1, 2021]. Available from: <https://wwwn.cdc.gov/Nchs/Nhanes/2017-2018/SMQ_J.htm>.

11. 2017-2018 Income (INQ_J) [Internet]. Centers for Disease Control and Prevention. 2021 [cited February 1, 2022]. Available from: <https://wwwn.cdc.gov/Nchs/Nhanes/2017-2018/INQ_J.htm>.

12. Ahluwalia N, Dwyer J, Terry A, Moshfegh A, Johnson C. Update on NHANES Dietary Data: Focus on Collection, Release, Analytical Considerations, and Uses to Inform Public Policy. Adv Nutr. 2016;7(1):121-34. Epub 2016/01/17. doi: 10.3945/an.115.009258. PubMed PMID: 26773020; PubMed Central PMCID: PMCPMC4717880.

13. Floegel A, Kim D-O, Chung S-J, Song WO, Fernandez ML, Bruno RS, et al. Development and validation of an algorithm to establish a total antioxidant capacity database of the US diet. Int J Food Sci Nutr. 2010;61(6):600-23. doi: 10.3109/09637481003670816.

14. Yang C, Jia X, Wang Y, Fan J, Zhao C, Yang Y, et al. Association between Dietary Total Antioxidant Capacity of Antioxidant Vitamins and the Risk of Stroke among US Adults. Antioxidants. 2022;11(11):2252.
